# Developing machine learning algorithms for meteorological temperature and humidity forecasting at Terengganu state in Malaysia

**DOI:** 10.1038/s41598-021-96872-w

**Published:** 2021-09-23

**Authors:** Marwah Sattar Hanoon, Ali Najah Ahmed, Nur’atiah Zaini, Arif Razzaq, Pavitra Kumar, Mohsen Sherif, Ahmed Sefelnasr, Ahmed El-Shafie

**Affiliations:** 1 College of Technical Engineering, Islamic University, Najaf, Iraq; 2 College of Engineering, Universiti Tenaga Nasional (UNITEN), 43000 Kajang, Selangor Malaysia; 3 Institute of Energy Infrastructure (IEI), Universiti Tenaga Nasional (UNITEN), 43000 Kajang, Selangor Malaysia; 4 Department of Civil Engineering, College of Engineering, Universiti Tenaga Nasional (UNITEN), 43000 Kajang, Selangor Malaysia; 5 College of Science, Al Muthanna University, Samawah, Al-Muthanna Iraq; 6 Department of Civil Engineering, Faculty of Engineering, Universiti Malaya (UM), 50603 Kuala Lumpur, Malaysia; 7 National Water and Energy Center, United Arab Emirates University, P.O. Box 15551, Al Ain, United Arab Emirates; 8 Civil and Environmental Engineering Department, College of Engineering, United Arab Emirates University, P.O. Box 15551, Al Ain, United Arab Emirates

**Keywords:** Atmospheric science, Hydrology

## Abstract

Accurately predicting meteorological parameters such as air temperature and humidity plays a crucial role in air quality management. This study proposes different machine learning algorithms: Gradient Boosting Tree (G.B.T.), Random forest (R.F.), Linear regression (LR) and different artificial neural network (ANN) architectures (multi-layered perceptron, radial basis function) for prediction of such as air temperature (T) and relative humidity (Rh). Daily data over 24 years for Kula Terengganu station were obtained from the Malaysia Meteorological Department. Results showed that MLP-NN performs well among the others in predicting daily T and Rh with R of 0.7132 and 0.633, respectively. However, in monthly prediction T also MLP-NN model provided closer standards deviation to actual value and can be used to predict monthly T with R 0.8462. Whereas in prediction monthly Rh, the RBF-NN model's efficiency was higher than other models with R of 0.7113. To validate the performance of the trained both artificial neural network (ANN) architectures MLP-NN and RBF-NN, both were applied to an unseen data set from observation data in the region. The results indicated that on either architecture of ANN, there is good potential to predict daily and monthly T and Rh values with an acceptable range of accuracy.

## Introduction

Air temperature (T) and relative humidity (Rh) are important in microclimate and environmental health research. It plays an essential role in several fields such as weather control, climate influence assessment of agricultural and water systems management. With the current global climate change, there is a need to develop a reliable model capable of accurately capturing the temperature and humidity changes. Many researchers have been developed models for predicting the meteorological time series based on statistical processes^1,2^. The clear problem is that these processors could not cope with specific non-stationary signals (time-series) and with signals whose mathematical model isn’t linear^3^. The difficulty is ascribed to the perspicuous environmental stochastic variables (meteorological processes such as temperature and relative humidity parameters) and the fact that future returns cannot be forecasted with acceptable precision when modelling such settings with high uncertainty.

Several forecasting models based on the univariate auto-regressive moving average representative of temperature and relative humidity was developed^4^. However, these models have a tendency to overestimate low temperature and relative humidity values while underestimating high values, which could lead to poor water resources planning and management. This is due to the fact that the meteorological processes are essential in estimating numerous hydrological parameters and irrigation processes including evaporation, evapotranspiration, infiltration, crop water requirement, and soil moisture, which are important for water resources managers and planners^5,6^. As a result, it is critical to creating a forecasting model that is both reliable and devoid of these shortcomings.

Temperature and relative humidity are thought to be extremely spatially distributed, time-varying, stochastic, nonlinear, and difficult to modelled utilizing simple models^7^. While conceptual models are important for understanding meteorological processes, these models experienced many practical circumstances especially when accuracy is the ultimate focus^8^. Instead of building a conceptual model, it could be superior to explore and employ different modelling approach such as data-driven model. Models based on differential equations are employed in the data-driven approach to detect the optimal inputs-outputs mapping deprived of a comprehensive examination of the fundamental configuration of phenomena process. In many areas, data-driven models have been shown to offer accurate predictions^9–11^. However, because most of these models do not attempt to reflect the nonlinear dynamics that are inherent in meteorological phenomena, they may not always perform well and achieve an acceptable level of forecasting accuracy as expected.

Machine learning modelling approaches have been proposed as an alternate modelling method for nonlinear and dynamic systems due to the fact that the machine learning approaches include effective structure and parameter estimation methodologies^12,13^. Since the characteristics of meteorological air temperature and relative humidity, it complex and nonlinear, machine learning (ML) models are powerful when implemented to problems whose resolutions required knowledge that is hard to specify. Unlike the conventional methods to time series analyses and predicting, ML models require a decreased quantity of information to predict the future time series. Based on the available time series, utilizing a suitable tuning algorithm, the internal network parameters are tuned. If necessary, this could also be including the adjustment of the primarily selected network structure to better matching the structure needs via the problem at hand^14^. Therefore, Machine learning approaches could be considered as an effective and efficient technique to model meteorological processes, based on their effectiveness in modelling dynamic systems in a variety of applications of science and engineering. The success of this strategy in cases when explicit knowledge of the internal meteorological phenomena is not available is its main advantage. In circumstances when modelling of the entire and/or part of the internal parameter of the meteorological phenomena is not available, machine learning modelling methodologies clearly give a realistic and effective approach for constructing input–output forecasting models. Despite the fact that those models have proven to be effective, it is still unknown which of these machine learning modelling approaches would be the best choice for certain system processes like meteorological processes. As a result, several machine learning modelling approaches must be examined in order to evaluate compare their performance, and hence, select the optimal one.

Recently, in meteorological time series Gradient Boosting tree was introduced as an easily adaptable machine learning approach in various aspects, such as filling gaps produced via missing or incorrect data^15^. G.B.T. has applied trend prediction of the asphalt pavement temperature accurately and has great ability in exploring the relationship between the temperature of asphalt pavements and meteorological factors^16^. In predicting and analyzing net ecosystem carbon exchange^17^ and modeling the energy consumption of commercial buildings G.B.T.^18^ was introduced. It is also recommended for daily accurate of reference evapotranspiration estimating in various climatic areas of China and somewhere else with the same climates around the globe^19^.

The R.F. approach's major advantages are its capability to generalize, lower sensitivity to parameter values, and built-in cross-validation. The R.F. technique's ability in^20^ has been examined in simulation long-term monthly air temperature. The results showed that the Random Forest approach is superior compared with the other methods. Also, authors in^21^ used the R.F. model for monthly temperatures predicting Kuala Lumpur in Malaysia using historical time series data from 2000 to 2012. R.F.'s performance was compared with other methods, application instances confirmed good properties of the R.F., which has higher accuracy. In^22^ R.F. was introduced for downscaling daily mean temperatures in the Pearl River basin in southern China. R.F. was demonstrated that the model has higher efficiency when compared with some alternative models. This result indicated that the Random Forest model is a feasible tool for the statistical downscaling of temperatures.

Neural network models, MLP-NN and RBF-NN, have been remarkable developments in the number and variations of the models established and the models' theoretical understanding in the past few years. The authors in^23^ examined the Artificial Neural network in predicting minimal temperatures. They have applied MLP-NN architecture to model the predicting system and back-propagation algorithm to train the networks. Their results found that minimal temperatures could be predicted with an acceptable level of accuracy by employing MLP-NN architecture. ANN has been applied in predicting the air temperatures and relative humidity in a building to reduce energy utilization for air conditioning^24^. Furthermore, the neural network was employed to estimate the hourly dew point temperatures, dew point temperatures is the temperature at which water vapor in the air condenses into liquid as in^25^. Also, researchers in^26^ was applied ANN to predicting monthly mean ambient temperatures.

It should be noted here that this study is the first attempt to investigate the performance of the machine learning modeling to predict the Temperature (T) and the Relative Humidity (Rh) in Terengganu, Malaysia, and hence, it is necessary to examine the performance of different machine learning modeling methods. Therefore, it is essential examine the potential of not only the “new” advanced machine learning modeling methods but also it is preferable to examine the “old” classical methods. In this context, in this study, two relatively new machine learning modeling methods including Gradient Boosting Tree (G.B.T.) and Random forest (R.F.) and two classical including Multi-Layered Perceptron (MLP) and Radial Basis Function (RBF) have been investigated. In fact, it is not mandatory that the advanced machine learning modeling methods outperformed the classical ones as the architecture and the mathematical procedure of the classical ones might be more suitable to detect the interrelationship between inputs and the output for these two variables.

In the light of the research background that has been presented above, it is clear that these meteorological variables under this study (humidity and temperature) have been studied, and several modeling approaches have been developed to predict them, including the utilization of different machine learning methods. However, in the development of these previous models, the model structure was almost the same using classical input pattern in the time series of these two variables. The existing models missed one major step in developing a successful prediction model, proposing different input–output mapping scenarios to detect the most appropriate model’s input(s) patterns. Therefore, in this study, twenty different input combination scenarios have been developed with respect to output patterns for a better choice of the most sensitive input(s) affecting the value of the desired output. In addition to that, most previous studies focused on monthly predicting. However, in the current study, the proposed models' reliability will be examined to capture daily and monthly fluctuations in the humidity and the temperature. Such two significant steps are the major novelty in the current research and could be considered the original research contribution in predicting the humidity and temperature, which could be considered a further step in the meteorological modelling.

To accomplish this goal, different artificial neural network (ANN) architectures (multi-layered perceptron, radial basis function was implemented; then it was compared with the Gradient Boosting tree (G.B.T.) and Random Forest (R.F.) techniques to predict daily and monthly air temperatures and relative humidity based on collected data from the year of 1985 to 2019.

## Material and methods

### Case study region and meteorological data

**Figure 1:**
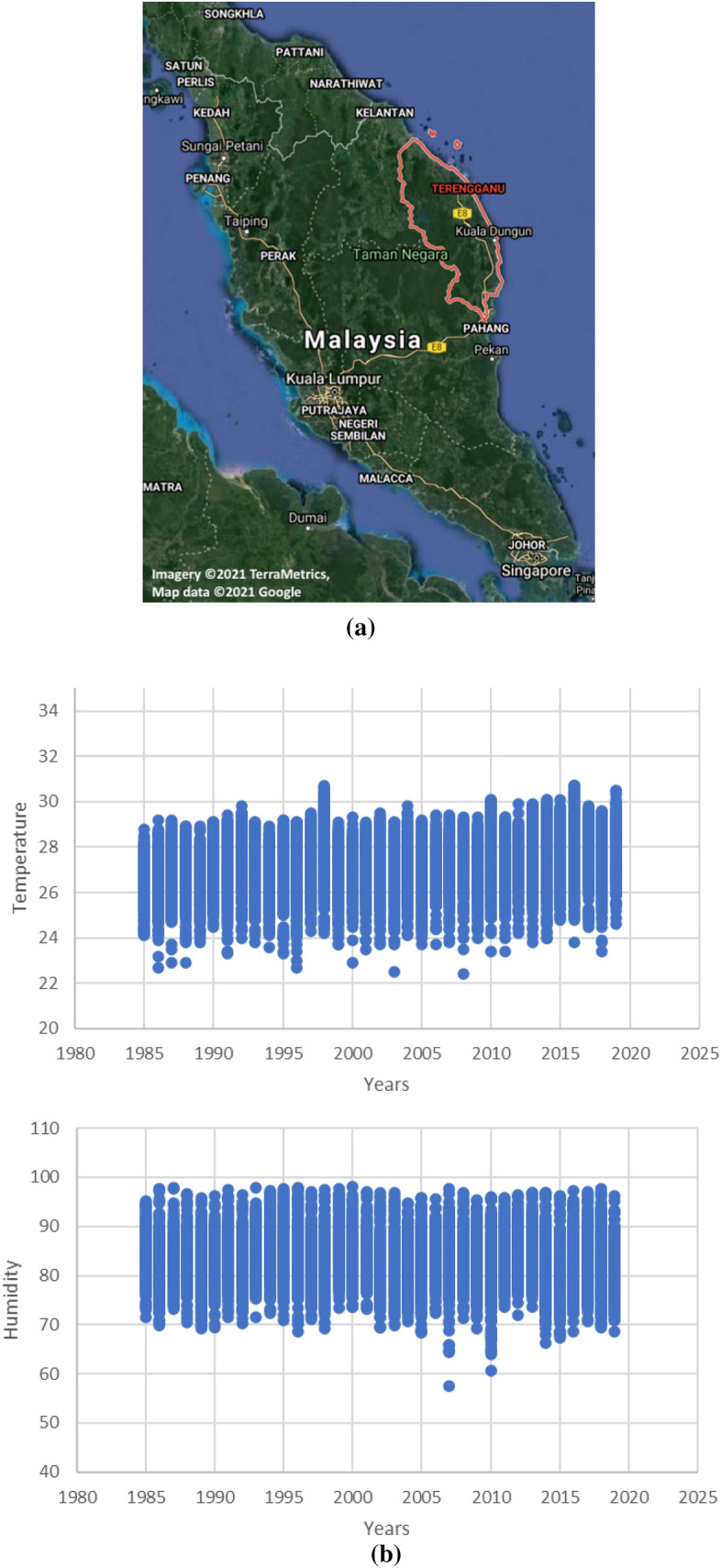
(**a**) Location of Kuala Terengganu on the map [Imagery ©2021 TerraMetrics, Map data ©2021 Google], (**b**) Temperature and humidity data.

The site is located in Kuala Terengganu, Malaysia and covers the largest city in the area^27^ at latitude 5° 23′ N and longitude 103° 06′ E. Figure 1 shows the location of the study area. Figure 1a has been generated by using Google Map software to identify the location of the study area. Air temperature and relative humidity data is observed and collected to predict them in Kuala Terengganu station accurately and is obtained from the Malaysian Meteorological Department. The observed and collected T and Rh data for the period years from 1985 to 2019. The data from 1985 to 2012 will be used to train the models, and from 2013 to 2019 will be used to test the proposed models. Table 1 shows the simple statistics done to ensure the data. It can be noticed that the height mean of daily air temperature is 30.7 °C highest, and relative humidity is 98.2%. In contrast, mean annual air temperature and relative humidity is 27.41 (°C) and 82.64%, respectively.

**Table 1:** Simple statistical analysis for the measured air temperature (T) and relative humidity (Rh).

	*T* (°C)	Rh %
Mean	27.20	82.74
Standard error	0.01	0.04
Median	27.20	82.50
Mode	27.10	83.30
Standard deviation	1.15	5.00
Sample variance	1.31	25.01
Kurtosis	57.85	42.89
Skewness	− 2.54	− 2.38
Maximum	30.70	98.20
Count	12,784	12,784

### Gradient boosting tree (G.B.T.)

Gradient boost tree will be used in this study regression model, an ML approach for regression problems in that the key predicting approach is a combining of some weak predicting models. G.B.T. approach is based on gradual strengthening of the predicting function $F_{b}$, via adding of the estimator $S$. The learning process is when the *S* is fitting to $\left( {y - F_{b} } \right)$ error (residual) and throughout every alteration $F_{b} + 1$ is adjusting to minimizing error values^28^:

1 $$F_{b + 1} \left( x \right) = F_{b} \left( x \right) + S = y - F_{b} \left( x \right)$$

For this objective, the loss function or ${\Psi }\left( {y,F\left( x \right)} \right)$ is specified and a series of inputs variables or $x = \left\{ {x_{1} , \ldots x_{n} } \right\}$ and a series of outputs values or $y$ are taken into account. A predicting modelling initiate via calculating the $F_{z} \left( x \right)$ as following:

2 $$F_{z} \left( x \right) = arg\;minimum_{\delta } \mathop \sum \limits_{\iota = 1}^{n} {\Psi }\left( {y_{\iota } ,\delta } \right)$$

**Figure 2:**
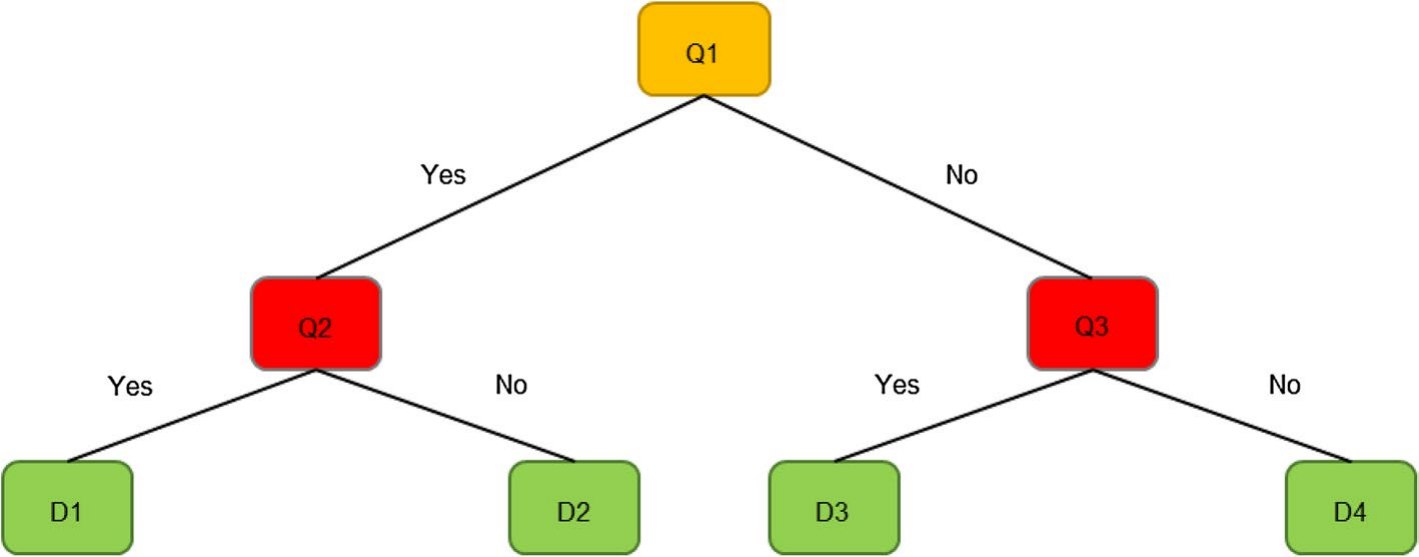
G.B.T approach.

Here, the $b{\text{th}}$ pseudo-residual value for $\iota {\text{th}}$ data set,$\delta_{\iota b}$ computed via: $\delta_{\iota b} = - \left[ {\frac{{\partial \psi \left( {y_{\iota } ,F\left( {x_{\iota } } \right)} \right)}}{{\partial \left( {F\left( {x_{\iota } } \right)} \right)}}} \right]F\left( {X_{\iota } } \right) = F_{b - 1} \left( {x_{\iota } } \right)$ for $\iota = 1, \ldots n$ Subsequently, the weak trainer function like a decision tree $\left( {E_{b} \left( {x_{\iota } } \right)} \right)$ is fitting to $\delta_{\iota b}$ and training based on the $\left\{ {\left( {x_{\iota } ,\delta_{\iota b} } \right.} \right\}_{\iota = 1}^{n}$ training sample. Through resolving a one-dimensional optimization relationship, the multiplier $\delta_{b}$ is computed as following:

3 $$\delta_{b} = arg\;minimum_{\delta } \mathop \sum \limits_{\iota = 1}^{n} {\Psi }\left( {y_{\iota } ,F_{b - 1} \left( {x_{\iota } } \right) + \delta h_{b} \left( {x_{\iota } } \right)} \right)$$

where $h_{b}$ is a new tree, the $F_{b} \left( {X_{\iota } } \right)$ a function is then taken = $F_{b - 1} \left( x \right) + \delta_{b} S_{b} \left( x \right)$ and the process is repeating till the second term of the total,$\delta_{b} S_{b} \left( x \right)$ is minimize at iteration $B$ where the final output $F_{B} \left( {x_{\iota } } \right)$ is attained, as shown in Fig. 2. For further details, more explanation can be found in^29,30^.

### Random forest (R.F.)

**Figure 3:**
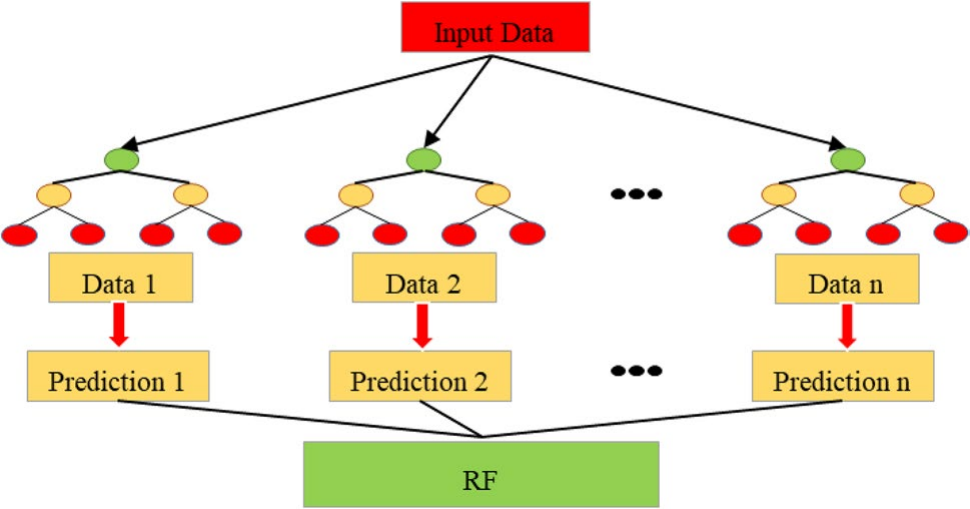
Structure of random forest (R.F.).

R.F. is a tree-based ensemble method, as shown in Fig. 3. It’s the algorithm that learns high-dimensional data. Every tree depends on collecting random variables, whereas, from several regression trees, a forest is growing to put with each other and forming an ensemble^31^. The bias is the same in all the trees, but the variances can be reduced by decreasing the relationship's coefficient^32^. Random forest for regression is dependent on a random vector, and it is created via grown trees, the trees predictor $d\left( {x,\varphi } \right)$ get numerical amounts. An outputs are numerical values, its assumed that the training sample is statistically independent. The mean square generalizing error of numerical predictor $d\left( x \right)$ could be expresses as following:

4 $$E_{X,Y} \left( {Y - d\left( x \right)} \right)^{2} = arg\;minimum_{\delta } \mathop \sum \limits_{i = 1}^{k} {\Psi }\left( {y_{i} ,F_{m - 1} \left( {x_{i} } \right) + \delta d_{m} \left( {x_{i} } \right)} \right)$$

The R.F. forecaster is created via averaging over $k$ of the trees $\left( {d\left( {x,\varphi } \right)} \right)$. More details regarding random forest model theories could be discovered in^31^.

### Artificial neural network (ANN)

It’s a computational model of the biological brain. It is included a considerable number of interconnecting neurons similar to the brain. Any neuron can perform just easy computations. However, the architectures of ANN are more simple comparing with a biological neuron. ANN is constructed in layers connecting to either single or many hidden layers where the actual processes is performed by weighted connection. In the hidden layers, any neuron connects to each neuron in the output layer. The result of the processing is obtained from the output layer. Learned ANN can attain via a certain training or learning algorithm that expands according to the learning law, supposed to mimic the biological system's learning mechanism^33^. Anyhow, as an assembly of neurons, in order to perform a complex task, ANN could be learning and involving: patterns recognitions, systems identifications, trend predicting, and processes controlling^34^.

#### Multilayer perceptron neural network (MLP-NN)

**Figure 4:**
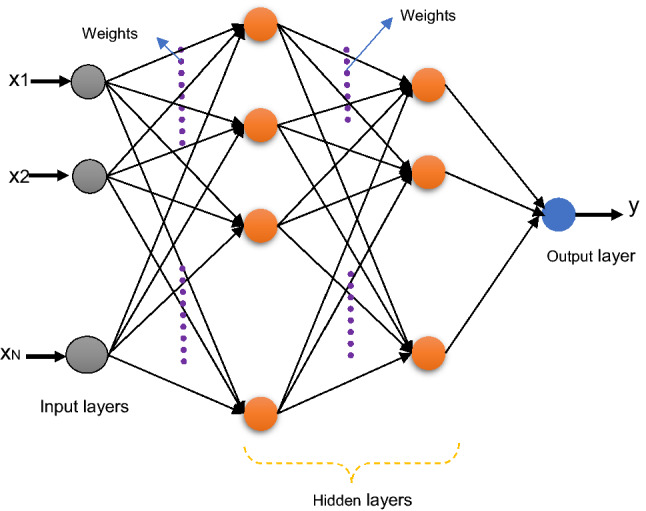
MLP-NN architecture.

In feedforward network Multilayer perceptron is maybe consider the most popular type. Figure 4 displays the M.L.P. with two hidden layers, input, and output layers. Only neurons acts as buffers in the input layer for the distribution of the signals of the input $x_{\iota }$ to neurons that are existing in hidden layers. In the hidden layers, any Neuron $j$ sum it is signals of the input $x_{\iota }$ after weighted them with the strength of the particular connection $wj_{\iota }$ from input layer, then calculates it is output $y_{j}$ which is consider as a function $f$ of sums up, as follows:

5 $$y_{j} = f \left( {\sum {wj_{\iota } x_{\iota } } } \right)$$

where $F$ could be an RBF or hyperbolic tangent or a sigmoidal or simple threshold function. In the output layer, there is a similar calculation for the output of the neuron. The most common training algorithms adopt back-propagation and gradient descent in Multilayer perceptron. It provides a changing $\Delta {\text{wj}}_{{{\iota }}}$ weight of the connections among neurons $\iota$ and ${\text{j}}$:

6 $$\Delta wj_{\iota } = \eta \delta_{j} x_{\iota }$$

Here $\delta_{j}$ is the factor that depends on whether neuron $j$ is the input neuron or a hidden neuron and $\eta$ is a parameter known as the learning rate. So, for output neuron:

7 $$\delta_{j} = \left( {\frac{\partial f}{{\partial net_{j} }}} \right)\left( {y_{j}^{\left( t \right)} - y_{j} } \right)$$

And for hidden neuron:

8 $$\delta_{j} = \left( {\frac{\partial f}{{\partial net_{j} }}} \right)\mathop \sum \limits_{q} w_{qj} \delta_{q}$$

In Eq. (7), $net_{j}$ is an overall weighted total of signals in the input layer to ($j$, $y_{j}^{\left( t \right)}$) neuron is the goal output for neurons $j$. For hidden neurons, there aren’t target outputs. As in Eq. (8), a variation of a desired and factual output of neurons in hidden layers $j$ which are replace via weighted sums of $\delta_{q}$ term previously achieved for neuron $q$ connecting to the output of $j$. Therefore, repeatedly, with output layer starting $\delta$ term for neuron is calculated in every layer then for every connection the weights updated defined. The weights updated processing could occur later than presenting every training pattern or following presenting of entire sets of training patterns. For both cases, a training epoch is considered complete when each training pattern was introduced once to Multilayer perceptron. In even the most trivialities, the M.L.P. must be adequately trained for several epochs. Adding the term momentum consider a standard method for accelerating learning to Eq. (6), allowing the change of weight to influence a new change in weight as below effectively:

9 $$\Delta wj_{\iota } \left( {k + 1} \right) = \eta \delta_{j} x_{\iota } + \mu \Delta wj_{\iota } \left( k \right)$$

Here $\mu$ represent a ‘momentum’ coefficient, whereas the $\Delta wj_{\iota } \left( {k + 1} \right)$, $\Delta wj_{\iota } \left( l \right)$ is the change of weights in epochs $\left( {k + 1} \right)$ and $\left( k \right)$ respectively.

#### Radial basis function neural network (RBF-NN)

**Figure 5:**
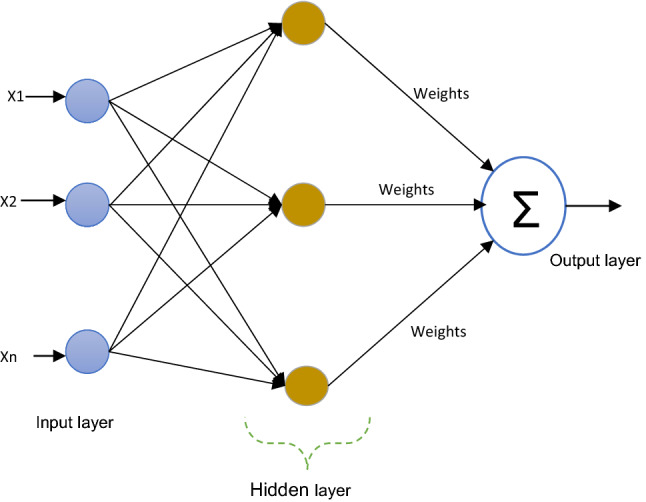
Architecture of RBF-ANN.

RBF neural network is a typical kind of ANN. Figure 5 shows the RBF-NN structure, which involves input, an output layer, and neurons' hidden layers. An input layer neuron received an inputs pattern ($x_{1}$ − $x_{N}$). Whereas a hidden layer neuron provided the sets of activation function, which represent the arbitrary “basis” for input pattern in an input range or space in order to expand in hidden range via manner of nonlinear conversion. A distance is computed between centre of every basis function and the input vector at the input of every hidden neuron. Using activation function to such distance produced the output of hidden neurons. The output of the Radial basis function neural network $y_{1}$ to $y_{p}$ are forming through neuron in output layer as weighted sum up of a hidden layer neurons basis. Usually, the activation function was selected as a standard function that’s positive at it is centre $x$ equal to zero, and then uniform declines to 0 on both sides. A popular option is a Gaussian function:

10 $$k\left( x \right) = exp\left( { - \frac{{x^{2} }}{2}} \right)$$

The function above could be shifting to a random centre $x$ equal to $m$, and stretched via varying it is spread $\sigma$ as following:

11 $$k\left( {\frac{x - m}{\sigma }} \right) = exp\left( { - \frac{{\left( {x - m} \right)^{2} }}{{2\sigma^{2} }}} \right)$$

The output of RBF-NN: $y_{j }$ is presented as below equation

12 $$y_{j} = \mathop \sum \limits_{\imath = 1}^{h} wj_{\imath } k\left( {\frac{{ \left\|x - m_{\imath }\right\| }}{{\sigma_{\imath } }}} \right)_{\forall x}$$

Here $wj_{\imath }$ represent weights of hidden neurons $\imath$ to $j$ which mean the output, $m_{\imath }$ the centre of activation function $\imath$, whereas $\sigma_{\imath }$ a spread of function $\left\|x - m_{\imath }\right\|$ is a norm of $x - m_{\imath }$. The norm could compute in many methods, but the most popular is the Euclidean norm defines as:

13 $$\left\|x - m_{\imath }\right\| = \sqrt {\left( {x_{1} - m_{\imath } } \right)^{2} + \left( {x_{2} - m_{\imath 2} } \right)^{2} + \cdots + \left( {x_{n} - m_{\imath n} } \right)^{2} }$$

This norm is giving the distance between point $x$ and point $m_{\imath }$ in N-dimension space. Every point $x$ that’s the same radial distance from $m_{\imath }$ gives a similar value to the norm. Learning (Training) RBF-NN aims to define the neuron weights $wj_{\imath }$, RBF center $m_{\imath }$ and spreads $\sigma_{\imath }$ which allow the network to produce the correct output $y_{j}$ related to the inputs pattern $x$.

### Statistical evaluation

In general, there is a need to examine the performance of the developed prediction model and compare the performance achieved from different models through particular statistical index. However, it is essential to use several statistical indices due to the fact that there is a possibility that two or more models could achieve similar or nearly values for particular statistical index, and hence, it is challenging to confirm which model outperformed the others. It should be noted here that each statistical index evaluates the model from single angle of the well-fitting between the model outputs and the desired values. Therefore, it is advisable to examine the model against several statistical indices to fully evaluate the performance each model individually and carry out a robust comparison analysis between them in order to get a solid confirmation about the most appropriate modeling method.

Three measures of performance are using to evaluate the result^35^: (R) the coefficient of correlation which is range (− 1 to 1), relative error measured (RMSE) Root mean square error and mean absolute error (M.A.E.) which is ranged between zero < M.A.E. < ∞. Higher values of R indicate superior model performance, and lower RMSE and M.A.E. indicate great model performance. According to^36^ as R value equal to 1 is mean the perfect fit, R more than 0.75 is a very good fit, R equivalent (0.64 to 0.74) is a good fit, R = 0.5 to 0.64 is an acceptable fit, and R less than 0.5 is an unacceptable fit.

14 $$R = \frac{{\mathop \sum \nolimits_{i = 1}^{n} \left( {M_{{o,{\mathfrak{i}}}} - \overline{{M_{o} }} } \right)\left( {M_{{P,{\mathfrak{i}}}} - \overline{{M_{P} }} } \right)}}{{\sqrt {\left( {M_{{o,{\mathfrak{i}}}} - \overline{{M_{o} }} } \right)^{2} \mathop \sum \nolimits_{i = 1}^{n} \left( {M_{{o,{\mathfrak{i}}}} - \overline{{M_{o} }} } \right)^{2} } }}$$

15 $${\text{RMSE}} = \sqrt {\frac{1}{{\text{n}}}\mathop \sum \limits_{{{\text{i}} = 1}}^{{\text{n}}} \left( {M_{{{\text{o}},{\text{i}}}} - M_{{{\text{p}},{\text{i}}}} } \right)^{2} }$$

16 $${\text{M}}.{\text{A}}.{\text{E}}. = \frac{1}{n}\mathop \sum \limits_{i = 1}^{n} \left| {\frac{{M_{o,i} - M_{p,i} }}{{M_{o,i} }}} \right| \times 100{ }$$

Here; $M_{{o,{\mathfrak{i}}}} { }$ represents values of meteorological data in the current observed $\left( i \right)$, $M_{{P,{\mathfrak{i}}{ }}}$ mean the predict values, $\overline{{{\text{M}}_{o} }}$ represent an average value of actual ‘observations’ and $\overline{{{\text{M}}_{P} }}$ refer to the average values of prediction, and $N$ represent a number of the dataset.

The choice of different statistical indices such as the RMSE and R is due to the fact that RMSE is proposed to examine the variance of the residuals, while R is to examine the relative measure of fit. RMSE indicates the absolute fit of the model to the data–how close the observed data points are to the model’s predicted values, which is a good measure of how accurately the model predicts the response. Whereas R is a relative measure of fit and has the useful property that its scale is intuitive and could examine the trend-match between the model outputs and the desired actual values.

From the above, it is clear that these R and RMSE are totally different, first, in terms of the mathematical formulation to calculate them as presented in Eqs. (14) and (15), second, as each one evaluates different measure of the model output compared with the desired observed values. As a result, it is expected that if two or more models provide same level of accuracy against particular statistical index, both could provide completely difference performance according to the other statistical index.

Given the requirements of the machine learning approaches, the raw meteorological dataset will normalize and range from zero to one before put in models by use the below formula^19^:

17 $$M_{n} = \frac{{M_{i} - M_{min} }}{{M_{max} - M_{min} }}$$

where $M_{n}$ and $M_{i}$ represents the normalizing and raw training—testing dataset; $M_{max}$ and $M_{min}$ represents the maximum and minimal training—testing dataset.

Furthermore, Taylor diagrams (T.D.s) provide a graphical representation of predicting and observed datasets. In the present work, T.D. will utilize for investigating how the model has higher accuracy compared with ML models' alternative. Some specifications of the predicting and actual values are data could be merged into T.D.^37^. For instance, the standard deviation (S.D.), CC, and RMSE between predicting and observed data could be demonstrated in the Taylor diagrams.

In this paper, we will also apply the method recommended by^38^ for the uncertainty analysis, which is calculated via (2.5 $xl$, 97.5 $xu$) percent percentiles.

18 $$Bracketed\;by\;95PPU{ } = { }\frac{1}{h}{ }Count{ }\left( {h{|}xl{ } \le h{ } \le { }xu} \right)*{ }100$$

where $h$ represents the number of actual data for a testing phase, in Eq. (18), the rate of ‘Bracketed by 95PPU’ is greater or 100 percent at completely actual data for a testing phase is between $xl$ that mean value of lower 95PPU and $xu$ it means value of upper 95PPU.

## Result and discussion

**Figure 6:**
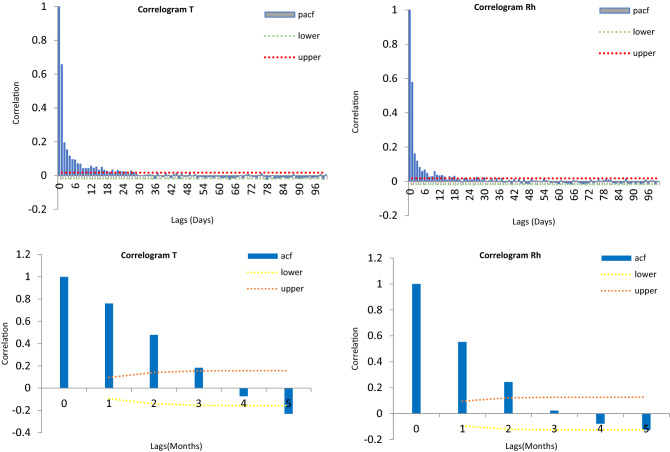
A.C.F. and PACF for air temperature and relative humidity using daily and monthly lags.

This paper's primary purpose is to evaluate machine learning models' performance (MLP-NN, RBF-NN, G.B.T., LR and R.F.) in predicting air temperature and relative humidity at Kuala Terengganu, Malaysia. To predict these meteorological data, quantify the level of correlation of data time series between output and input variables for different times. Two methods were applied to identify the optimal lag of antecedents’ predictor by assuming several lag times. The autocorrelation function (A.C.F.) is a statistical analysis used to evaluate the correlation among adjacent values correlation^39^. Simultaneously, the partial autocorrelation (PACF) is defined as the partial correlation with its lag values of a time series at the same time didn't consider the effects of intervening lag auto-correlation^40^. A.C.F. applied for monthly input design, while partial autocorrelation function used for daily input design as in Fig. 6. The historical meteorological dataset on T and Rh corresponding to various lags beginning from the original day (t) to 6 days earlier (time t–6) are using as daily input for the models to predicting meteorological data on day $t$ in Table 2. According to PACF, the highest correlation between input and output is moderate 0.66 and 0.58 for both $T_{t}$ and $Rh_{t}$ , there is a declining in correlation when increase daily time span between input and output (denotes t = daily time step) as in Fig. 6. Also, from Fig. 6 it could see the value of A.C.F. between the target time series $T_{m}$ and $T_{m - 1}$, $T_{m - 2}$, $T_{m - 3}$ and $T_{m - 4}$ (where m is month lag) is decreased as increase lag time, where the high correlation gets with 0.75 in lag 1 month. Thus, these input combinations time series are considered in this work as probable inputs to predicting the $T_{m}$ monthly time series. And same procedure accomplished for $Rh_{m}$ also better correlation is moderate with 0.5 between input and output relative humidity variable, and it was found there is a decrease in correlation as increase lag time so the input structures as in Table 2. To predict $Rh_{m}$. In contrast, the target time series T and Rh's dependence characteristics with previous times series of T and Rh have decreased trend via increase the lag time. Therefore, the input design is shown in Table 2.

**Table 2:** Input design for air temperature and relative humidity.

Name models	Input combination	Output
M1	Tt-1	Tt
M2	Tt-1, Tt-2	Tt
M3	Tt-1, Tt-2, Tt-3	Tt
M4	Tt-1, Tt-2, Tt-3, Tt-4	Tt
M5	Tt-1, Tt-2, Tt-3, Tt-4, Tt-5	Tt
M6	Tt-1, Tt-2, Tt-3, Tt-4, Tt-5, Tt-6	Tt
M7	Rh t-1	Rh t
M8	Rh t-1, Rh t-2	Rh t
M9	Rh t-1, Rh t-2, Rh t-3	Rh t
M10	Rh t-1, Rh t-2, Rh t-3, Rh t-4	Rh t
M11	Rh t-1, Rh t-2, Rh t-3, Rh t-4, Rh t-5	Rh t
M12	Rh t-1, Rh t-2, Rh t-3, Rh t-4, Rh t-6, Rh t-6	Rh t
M13	Tm-1	Tm
M14	Tm-1, Tm-2	Tm
M15	Tm-1, Tm-2, Tm-3	Tm
M16	Tm-1, Tm-2, Tm-3, Tm-4	Tm
M17	Rh m-1	Rh m
M18	Rh m-1, Rh m-2	Rh m
M19	Rh m-1, Rh m-2, Rh m-3	Rh m
M20	Rh m-1, Rh m-2, Rh m-3, Rh m-4	Rh m

After data analysis and normalization, the meteorological data are divided into sets, 80%, and 20%, for the training and testing phases, respectively. The models' predicting performance developed in this paper is evaluated based on the statistical evaluation (CC, MAE, and RMSE). Table 3 demonstrates the summary of the results obtained in this study of prediction performance indices for the predictive models MLP-NN, RBF-NN, G.B.T., R.F. and L.R.

**Table 3:** Results of a performance evaluation using the developed ML techniques during testing phase.

	MLP			RBF			GBT			RF			LR		
	R	MAE	RMSE	R	MAE	RMSE	R	MAE	RMSE	R	MAE	RMSE	R	MAE	RMSE
M1	0.695	0.019	0.027	0.691	0.019	0.027	0.692	0.019	0.027	0.694	0.019	0.027	0.656	0.019	0.027
M2	0.702	0.018	0.027	0.677	0.019	0.028	0.699	0.019	0.027	0.701	0.019	0.027	0.671	0.019	0.023
M3	0.707	0.018	0.026	0.435	0.032	0.036	0.704	0.018	0.027	0.703	0.019	0.027	0.678	0.019	0.026
M4	0.709	0.018	0.026	0.444	0.031	0.034	0.706	0.018	0.026	0.704	0.019	0.027	0.686	0.018	0.026
M5	0.710	0.018	0.026	0.488	0.024	0.033	0.709	0.018	0.026	0.697	0.019	0.027	0.686	0.018	0.026
M6	0.713	0.018	0.026	0.433	0.037	0.032	0.711	0.018	0.026	0.697	0.019	0.027	0.686	0.018	0.026
M7	0.609	0.027	0.040	0.591	0.027	0.041	0.597	0.028	0.041	0.611	0.027	0.040	0.575	0.027	0.040
M8	0.618	0.027	0.040	0.544	0.029	0.043	0.607	0.027	0.041	0.623	0.027	0.040	0.583	0.027	0.040
M9	0.624	0.027	0.040	0.458	0.029	0.029	0.610	0.027	0.040	0.627	0.027	0.040	0.592	0.027	0.040
M10	0.624	0.027	0.040	0.408	0.042	0.037	0.612	0.027	0.040	0.629	0.026	0.040	0.613	0.027	0.040
M11	0.625	0.026	0.040	0.434	0.039	0.035	0.613	0.027	0.040	0.631	0.026	0.040	0.614	0.027	0.040
M12	0.634	0.026	0.039	0.472	0.023	0.029	0.615	0.027	0.040	0.631	0.026	0.040	0.616	0.026	0.040
M13	0.767	0.013	0.016	0.784	0.013	0.016	0.782	0.013	0.016	0.776	0.013	0.016	0.735	0.013	0.016
M14	0.786	0.012	0.016	0.801	0.012	0.015	0.801	0.012	0.015	0.807	0.012	0.015	0.748	0.012	0.016
M15	0.828	0.011	0.014	0.830	0.011	0.014	0.818	0.011	0.015	0.840	0.011	0.014	0.768	0.012	0.015
M16	0.846	0.011	0.014	0.832	0.011	0.014	0.830	0.011	0.014	0.840	0.011	0.014	0.775	0.012	0.015
M17	0.582	0.018	0.023	0.614	0.017	0.023	0.639	0.017	0.022	0.618	0.017	0.023	0.528	0.018	0.023
M18	0.655	0.017	0.022	0.666	0.016	0.021	0.632	0.017	0.022	0.662	0.017	0.021	0.529	0.018	0.023
M19	0.670	0.016	0.021	0.703	0.016	0.020	0.646	0.017	0.022	0.702	0.016	0.021	0.536	0.018	0.023
M20	0.651	0.017	0.022	0.713	0.015	0.020	0.653	0.017	0.022	0.684	0.016	0.021	0.539	0.018	0.023

With regards to daily air temperature, for MLP-NN and G.B.T. methods, the optimal daily air temperature in prediction is obtained for input combination previous 6 days (M6) with good value of R equal to 0.713 and 0.711, respectively. At the same time, R.F. approach shows the best performance during a time horizon equal to 4 days (M4) with R equal to 0.704. The results regarding applying for the LR model list forth in the order of model performance where R value equal to 6.686 for three models (M4–M6). RBF exhibits the lowest performance compared with all other proposed models.

Regarding prediction daily relative humidity, the performance of LR model increase as increase input variables until it almost shows constant values with CC = 0.6 which is the lowest than values get from G.B.T, R.F. and MLP-NN. Also, it could be noticed that the RBF-NN approach did not perform well, where the highest value gets it for R is 0.59 and 0.54 using lead time 1 and 2 days of Rh values (M7 and M8), respectively. In contrast, M12 using the previous 6 days of Rh as input achieves better performance compared with other structures by applying MLP-NN and G.B.T. with a moderate level of accuracy in daily prediction Rh with R 0.6335 and 0.6152, respectively. The lowest values of MAE and RMSE are achieving at M12 with 0.0263 and 0.0394, respectively. The input combination M11 using the previous 5 days' values as input for the R.F. model demonstrates optimum performance with R found to be 0.63 and the lowest values for both M.A.E. and RMSE out of 0.026 0.039.

All monthly prediction air temperature methods achieved better performance for input structure previous 4 months values of T (M16) with good R of 0.846, 0.832, 0.83, and 0.84 for MLP-NN, RBF-NN, G.B.T., and R.F., respectively. Besides, over M16, MLP-NN produces a lower error with M.A.E. = 0.0105 and RMSE = 0.0135 compared to other techniques.

**Figure 7:**
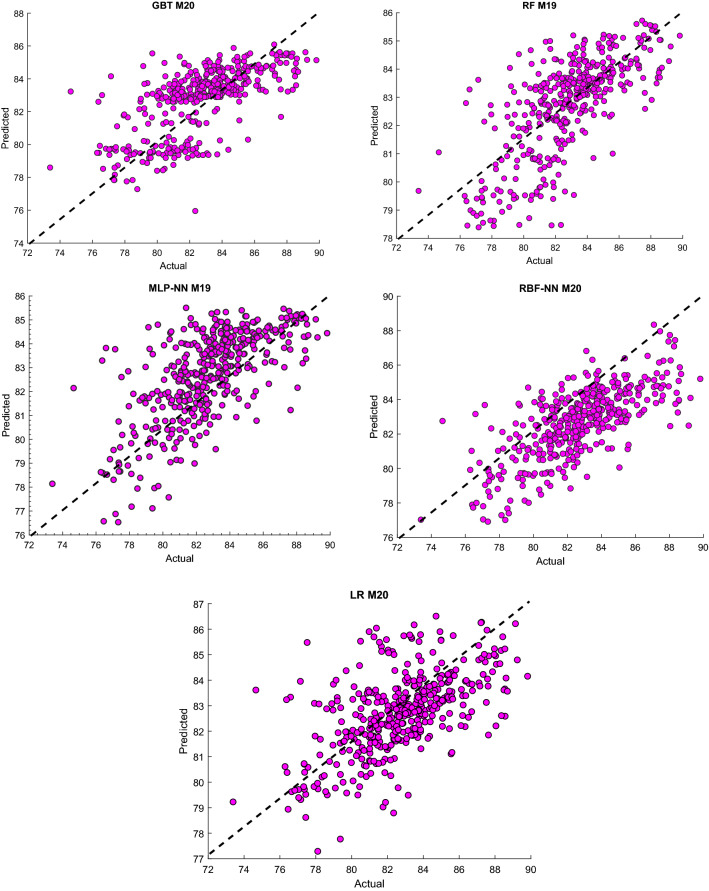
Scatter plots of actual and predicting monthly Rh use the most accurate combination of input parameters using the developed ML algorithms.

**Figure 8:**
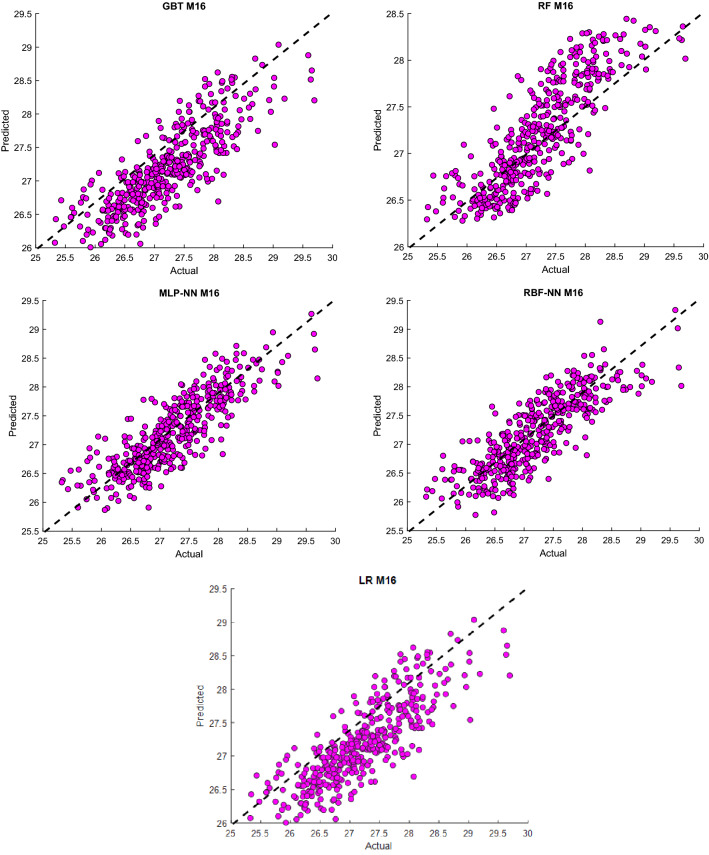
Observed and predicted monthly T values use the most accurate input combination using the developed ML algorithms.

Concerning monthly prediction relative humidity, the highest R with the lowest RMSE were obtained from the RBF-NN algorithm using the input structure previous 4 months of Rh (M20). Also, M20 was superior to the G.B.T. considerably in among all input combinations. At the same time, M19 showed the highest performance compared with other structures for MLP-NN and R.F., with R of 0.6704 and 0.7019, respectively. Finally, we could conclude that ANN-MLP, G.B.T. and R.F. model shows better performance than LR model in all input combination designs for prediction T and Rh in daily and monthly horizon. For better visualization, scatter plots between the actual values and predict T and Rh's value using the most accurate input combinations are shown in Figs. 7 and 8 for station Kuala Terengganu, Malaysia.

It could be observed that both modeling approaches LR and the machine learning models provide nearly same results considering the RMSE as the statistical index. On the other hands, it could be noted that the machine learning model outperformed the LR model while examining the R value as the statistical index. However, in general, there is a potential to improve the predicting accuracy for both variables T and Rh whether for daily or monthly time-increments. From this perspective, the machine learning as modeling approach is more convincer and has stronger potential for further improvement over the LR model to achieve better prediction accuracy for these two variables for both time-increments.

Although Figs. 7 and 8 and Table 3 show the observed and predicted values and evaluation criteria for all models, summarized the comparison results among ML models could not be discussed via these figures and table. Therefore, the Taylor diagram (T.D.) using the most accurate input structures from the above evaluation criteria will compare the techniques presented in this study. The main concept of the Taylor diagram is to presents the closest prediction model with the corresponding actual observation in the 2-dimensional scaling (standard deviation (S.D.) on the polar axis and the R on the radial axis)^37^. Standard deviation referring to how much on average measurements differ from each other. So, the relative value of standard deviation predicted (SDP) from standard deviation actual (S.D.A.) indicates high precision.

**Figure 9:**
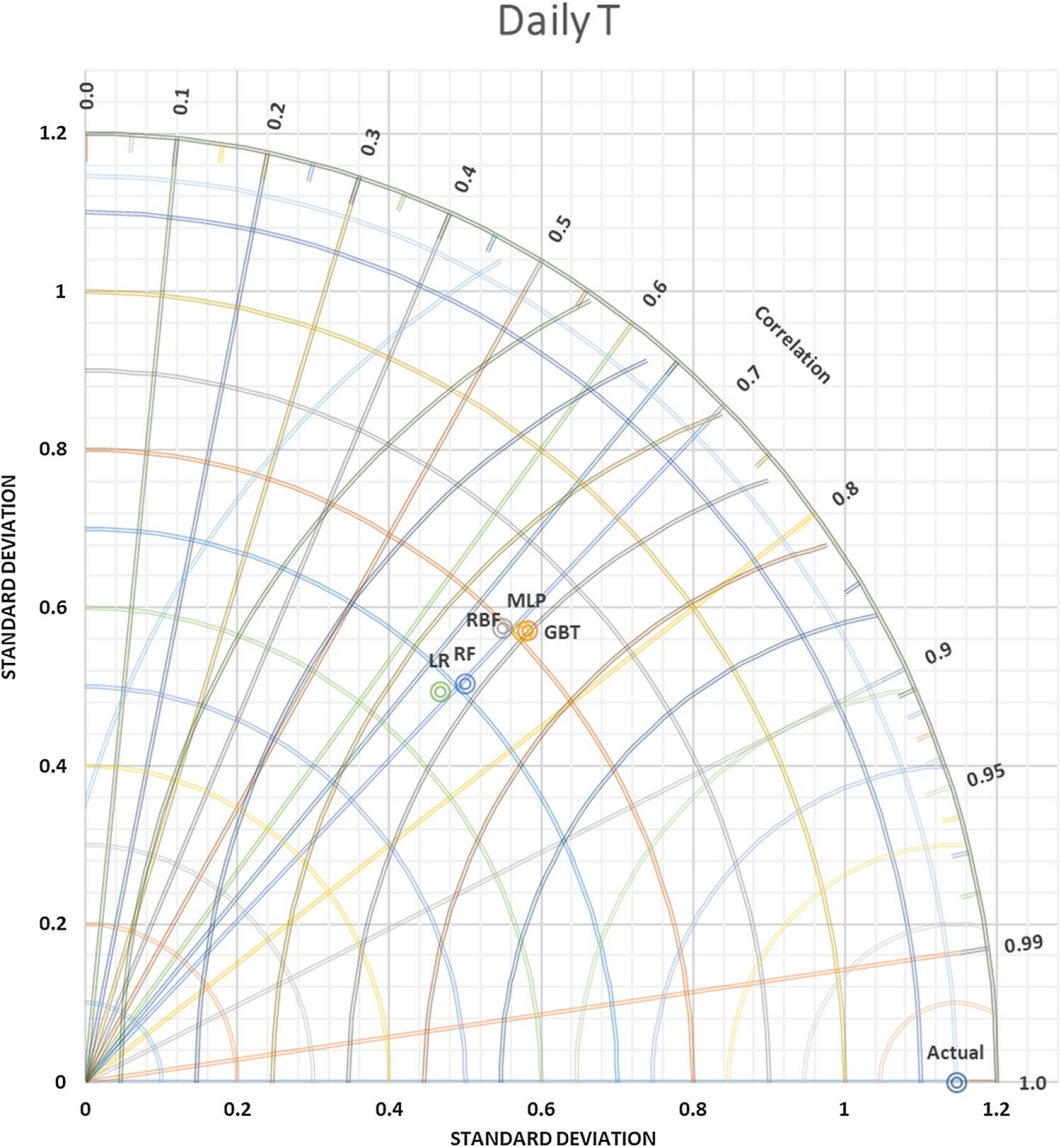
Taylor diagram of predicting daily T amounts using the most accurate models.

**Figure 10:**
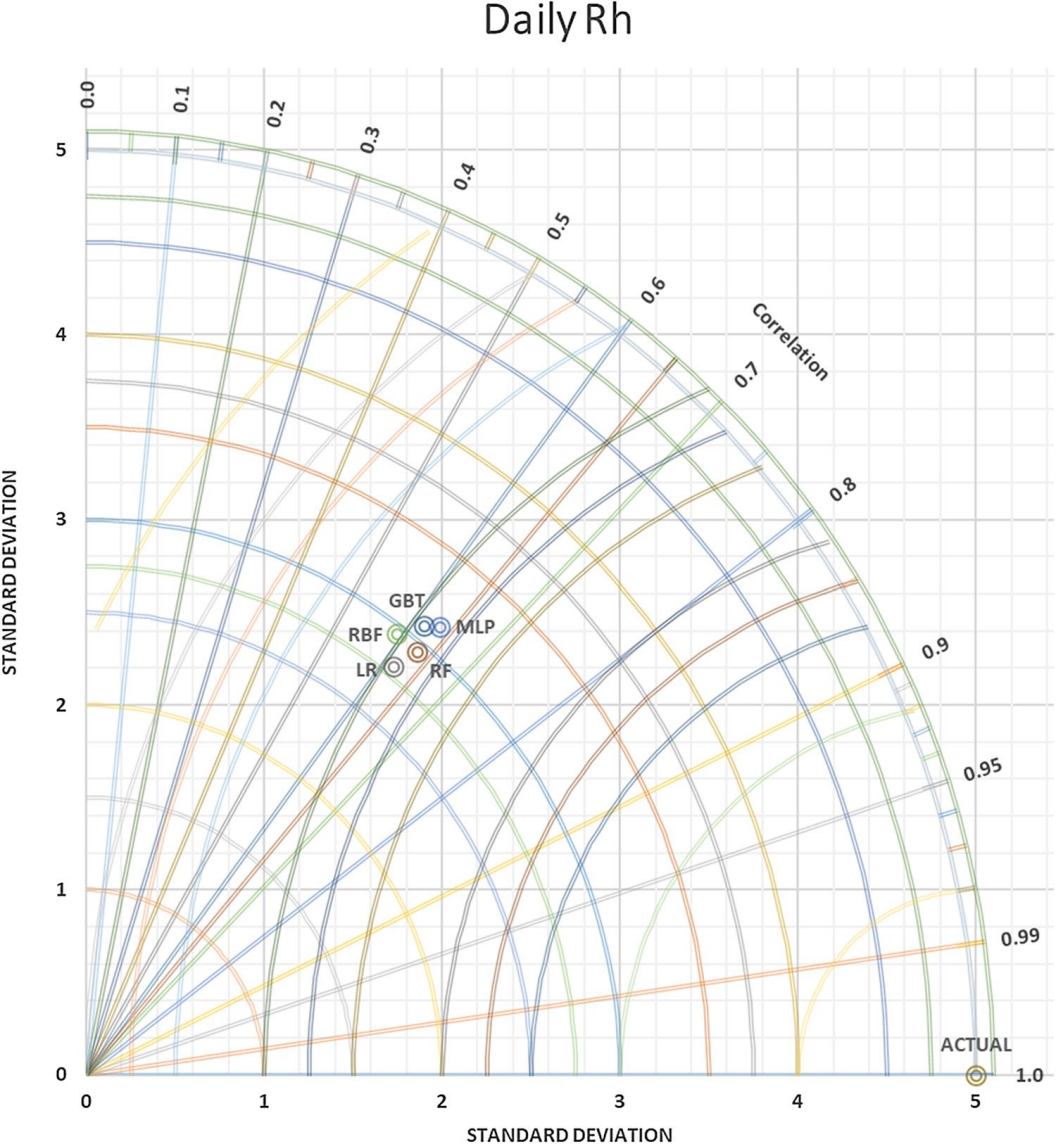
Taylor diagram of predicting daily Rh percent using the most accurate models.

**Figure 11:**
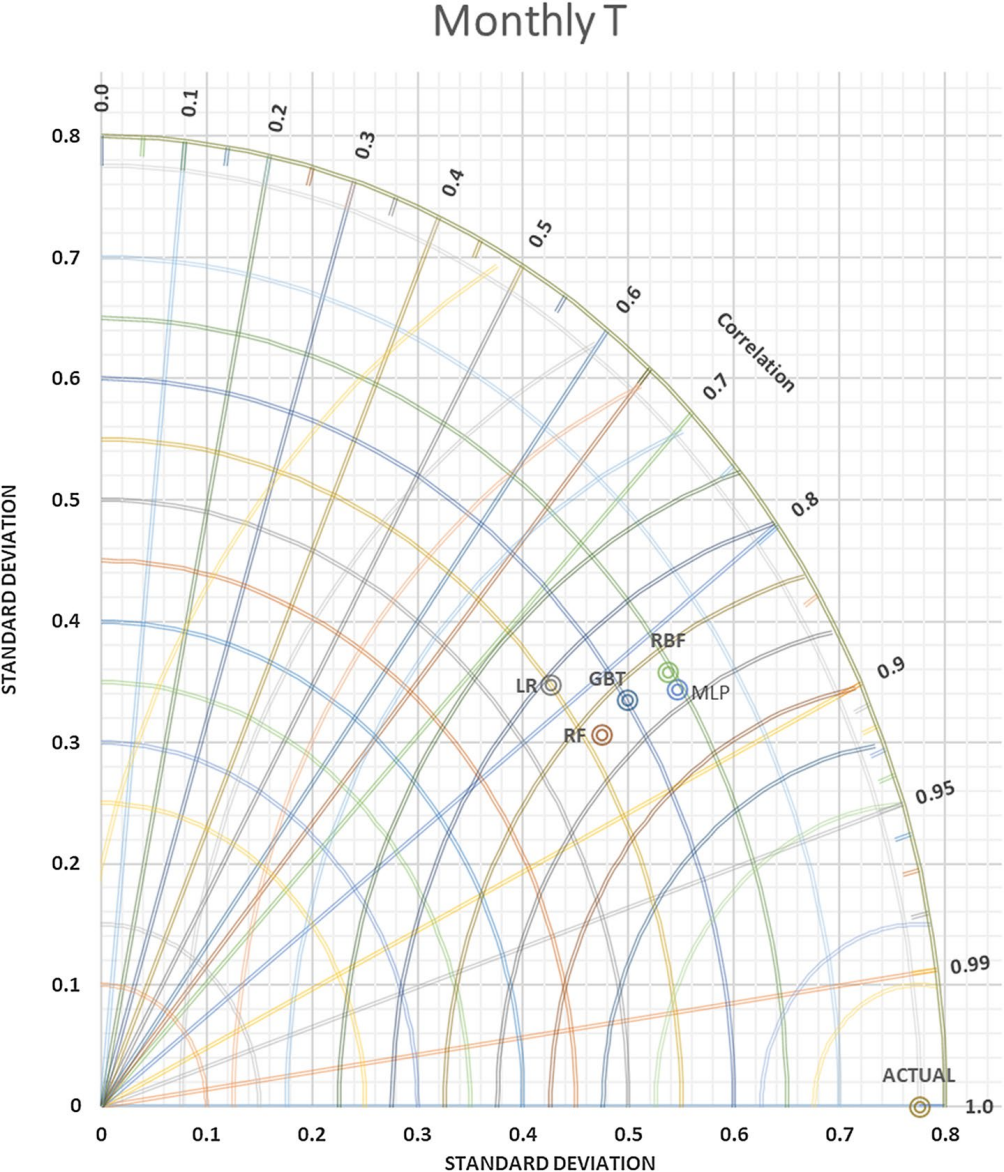
Taylor diagram of predicting monthly T amounts using the most accurate models.

**Figure 12:**
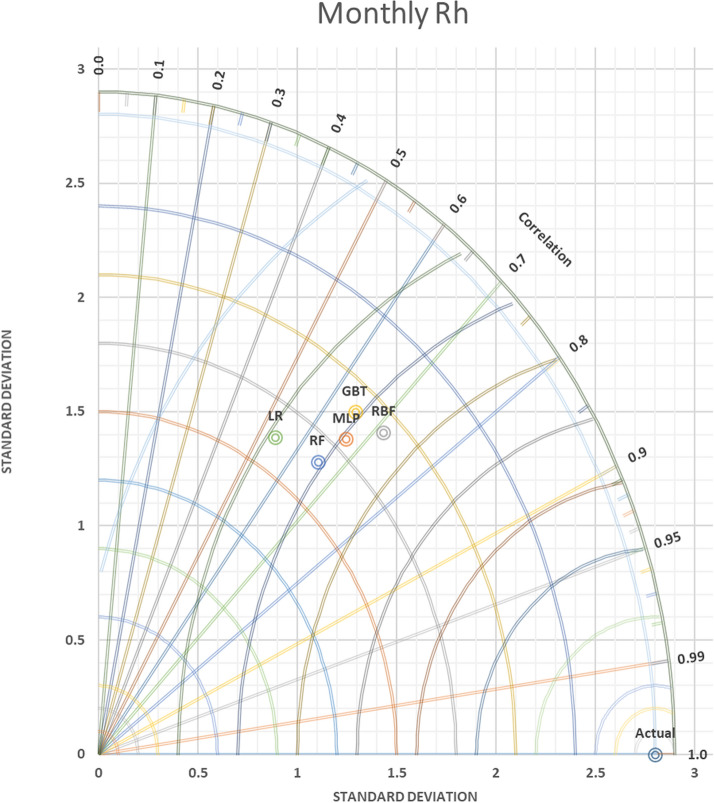
Taylor diagram of predicting monthly Rh percent using the most accurate models.

In contrast, versus as far the value of SDP from S.D.A. refers to lower accuracy. Thus, in Figs. 9 and 10 daily T and daily Rh, it can be observed that the MLP-NN was superior compared to other approaches, which have closer SD with 0.81486 to actual SD 1.146252 in daily T and SD = 3.134169 predicted daily Rh to actual SD = 5.001828. An evaluation of actual and predictor T monthly values yielded via the most accurate models was done for station Kuala Terengganu. T.D.s are shown for T in Fig. 11 that proved the slight efficiency of MLP-NN over RBF-NN, G.B.T., and R.F. models. As shown in Fig. 12, RBF-NN predicted Rh m values more accurately than other models with best SDP = 2.010736.

### Accuracy improvement (A.I.)

**Figure 13:**
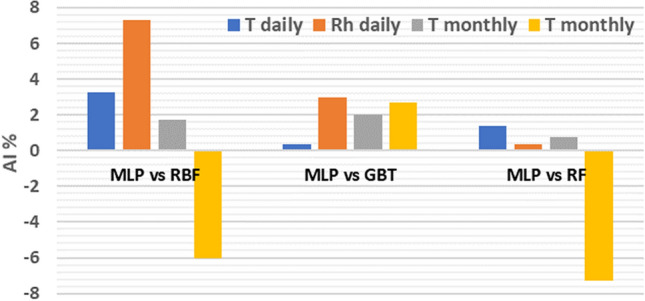
Summary of A.I. for MLP-NN over daily and monthly predict T and Rh.

In order to demonstrate the accuracy improvement, we calculated the accuracy improvement of the MLP-NN compared to RBF-NN, G.B.T. and R.F. using the A.I. indicator Eq. (19).

19 $$AI = \left( {\frac{{CC_{MLP} - CC_{n} }}{{CC_{n} }}} \right)*100$$

where $CC_{n}$ is the correlation coefficients for three machine learning model, and $CC_{MLP}$ is the correlation coefficient for the MLP-NN model. It can be seen from Fig. 13, a noticeable level of improvement has been achieved when MLP-NN is adopted compared to three other models (RBF-NN, G.B.T., and RF) for both parameters during the daily proposed time horizons. A.I. varies between 0.3% to more than 7%, where the highest A.I. achieved when the MLP-NN model was used to predict daily relative humidity. Even though MLP-NN also outperform with positive values over three models in prediction monthly T, but for prediction monthly Rh it showed negative value over RBF-NN and R.F. models with − 6% and − 7%, respectively as in Fig. 13 due to the MLP-NN has lesser values of R compare with RBF-NN and G.B.T. out of 0.6507, 0.7133, 0.6836, respectively.

### Uncertainty analysis

The prediction uncertainty of the proposed models was examined using the 95PPU. The d-factor (20), an indicator of the standard deviation interval's width, was also carried out.

20 $$d{ - }factor = { }\frac{{\overline{dx} }}{\sigma x}$$

where σ$x$ is the S.D. of observed data $X$, and $\overline{dx} { }$ is the average distance between the upper and lower bands, which could be computed using the below formula:

21 $$\overline{dx} = \frac{1}{k}\mathop \sum \limits_{i = 1}^{k} \left( {{ }XU{ } - { }XL{ }} \right)$$

The conclusions drawn from the analysis of two uncertainty measures can be seen in Table 4.

**Table 4:** Results of 95PPU and d-factor for best models in prediction daily and monthly.

	Model	Lower	Upper	95PPU	d-factor
**T**					
Daily	MLP-NN	24.62741	30.35311	99.9765	0.00039
Monthly	MLP-NN	25.9026	123.3846	99.5147	0.3037
**Rh**					
Daily	MLP-NN	50.31563	91.68713	99.8278	0.000647
Monthly	RBF-NN	77.0440	174.4773	99.0291	0.0842

The results show that about 100 percent of the predicted data is within the range of predicted values based on 95PPU, while the d-factors are low. Such results indicate the reliability of the proposed models in predicting the air temperature and relative humidity.

Recall that the purpose of the current research is to investigate the potential of the existing modeling approaches to provide accurate prediction accuracy for T and Rh for different time-increments. Although a simple equation based on the LR modeling approach could provide relatively similar accuracy of the machine learning model based on particular statistical index, it is worth to examine different statistical index to confirm and settle the effectiveness of all the examined models. In addition, as it could be noticed that the achieved accuracy from both model approaches are not the expected outstanding ones, it is essential to keep improving the better one, and hence, there is a need to figure out the modeling approach that has enough potential for further improvement over the others. On the other hand, for the first attempt applications of the machine learning modeling approaches, it is necessity to examine both the “new” advanced and “old” methods, which could be considered as the major value of the current research.

## Conclusions

The accuracy of ML models, namely MLP-NN, RBF-NN, G.B.T., L.R. and R.F., were investigated to predict air temperatures and relative humidity in different time horizons (daily and monthly) using historical meteorological data. Different input combinations were investigated with varying times of lag. The results showed that MLP-NN is a leading algorithm compared with other models in predicting monthly relative humidity with a correlation coefficient equal to 0.8462, followed by RBF-NN with a correlation coefficient equal to 0.7113. By applying Accuracy Improvement, 7% of improvement was achieved after proposing MLP-NN. Uncertainty analysis using 95PPU and d-factor was conducted to test the reliability of the MLP-NN model. It can be concluded that the proposed model can be adopted in predicting these two parameters, even with a new dataset. Although the developed MLP model's attained result is acceptable, future works can be explored by hybridizing the MLP-NN model with optimizers for more accuracy.
